# Forecasts of COPD mortality in Australia: 2006-2025

**DOI:** 10.1186/1471-2288-12-17

**Published:** 2012-02-21

**Authors:** Bircan Erbas, Shahid Ullah, Rob J Hyndman, Michelle Scollo, Michael Abramson

**Affiliations:** 1 School of Public Health, La Trobe University, Rm 129, Health Sciences 1, Bundoora, Victoria, 3086, Australia; 2 Flinders Centre for Epidemiology and Biostatistics, School of Medicine, Flinders University, South Australia 5042, Australia; 3 Department of Econometrics and Business Statistics, Monash University, Clayton 3800, Australia; 4 VicHealth Centre for Tobacco Control, The Cancer Council Victoria, 100 Drummond St, Carlton, Victoria 3053, Australia; 5 School of Public Health and Preventive Medicine, Alfred Hospital, Monash University, Melbourne 3004, Australia

**Keywords:** COPD mortality, Functional data analysis, Tobacco consumption, Forecasting

## Abstract

**Background:**

Chronic Obstructive Pulmonary Disease (COPD) is currently the fifth leading cause of death in Australia, and there are marked differences in mortality trends between men and women. In this study, we have sought to model and forecast age related changes in COPD mortality over time for men and women separately over the period 2006-2025.

**Methods:**

Annual COPD death rates in Australia from 1922 to 2005 for age groups (50-54, 55-59, 60-64, 65-69, 70-74, 75-79, 80-84, 85+) were used. Functional time series models of age-specific COPD mortality rates for men and women were used, and forecasts of mortality rates were modelled separately for men and women.

**Results:**

Functional time series models with four basis functions were fitted to each population separately. Twenty-year forecasts were computed, and indicated an overall decline. This decline may be slower for women than for men. By age, we expect similar rates of decline in men over time. In contrast, for women, forecasts for the age group 75-79 years suggest less of a decline over time compared to younger age groups.

**Conclusions:**

By using a new method to predict age-specific trends in COPD mortality over time, this study provides important insights into at-risk age groups for men and women separately, which has implications for policy and program development.

## Background

Chronic Obstructive Pulmonary Disease (COPD), including chronic bronchitis and emphysema, is currently the fifth leading cause of death in Australia. It was responsible for 31.2 male and 16.3 female deaths per 100,000 population (age-standardized) in 2005 [1].

The major known risk factor is tobacco smoking. However, there is a substantial latent period after the uptake of regular smoking, so that COPD typically only becomes manifest decades later. COPD is the only major condition for which the burden of disease (as measured at the population level) is continuing to increase with an ageing population, which is at a higher risk of developing COPD [2]. This is because COPD results in a progressive respiratory disability, a substantial impairment in the quality of life and frequent hospital admissions. COPD is also accompanied by substantial comorbidities, including depression, heart disease and osteoporosis. Yearly mortality time trends which are collected by the Australian Bureau of Statistics provide a sound basis for modelling the occurrence of COPD over the twentieth century and for predicting the likely situation over the next 20 years. A model which predicts future age-specific COPD mortality rates separately for men and women should also enable policy-makers to extrapolate the health care costs associated with treatment, and to calculate the probable economic benefit of policies which effectively eliminate smoking and control other risk factors.

In this study, we use functional data analysis (FDA) techniques to model age-specific time trends of COPD mortality rates for men and women in Australia, and to estimate future mortality rates over the next two decades. The use of models which also incorporate functional data analysis for forecasting mortality is relatively new. Hyndman and Ullah [3] proposed forecasting age-specific mortality rates which are observed over time. These forecasting models incorporate the entire age-mortality relationship over time, rather than using only the most recently available data. These models are an alternative to the age-period-cohort models which are commonly used to model and predict future mortality trends over time.

In this study we seek to: a) model age related changes in COPD mortality time trends separately for men and women using functional data analysis, and b) predict future age related changes in COPD mortality rates separately for men and women.

## Methods

### Data

The annual COPD death rates (1922-2005) for the age groups (50-54, 55-59, 60-64, 65-69, 70- 74, 75-79, 80-84, 85+) were obtained from the General Record of Incidence of Mortality (GRIM) books maintained by the Australian Institute of Health and Welfare [4]. These COPD mortality rates are defined as the number of deaths in a particular age group during the year, divided by the corresponding mid-year population. The rate is expressed per 100,000 people. Data for COPD (ICD10 J41-J44) are available from 1922 to 2005. All deaths have been mapped to ICD10 codes, in accordance with Taylor [5], and, more recently, the ICD (International Classification of Disease) 9-ICD10 mapping tables released by the World Health Organization.

#### Functional data analysis models

In this study, we use the annual COPD mortality rates as a function of age (taken to be the midpoint of the age groups). We plot the age and mortality for each year, and define these as mortality-age curves. We take the log of COPD mortality and use functional data analysis (FDA) techniques [6] to model these curves collectively as a functional time series. We first smooth the data for each year using a nonparametric smoothing method, in order to estimate these functions. As the mortality rates are functions of age, we assume that the estimable functions increase monotonically with age (where age is at least 50 years). To capture these trends, we use constrained penalized regression splines [7,8]. We define these smooth curves as our functional observations, and fit FDA models, as proposed by Hyndman and Ullah [3], to estimate the functions which represent the age-mortality relationship. Each curve is expressed as a linear function of nonparametric basis functions, defined using a principal component decomposition; in practice, we find that the use of about four or five basis functions is usually sufficient to capture the variations in the data. The basis functions do not vary with time, but the associated coefficients (the principal component scores) form time series which can be forecast. This separation of age effects (the basis functions) and time effects (the coefficients) enables the age-specific mortality rates to be forecast into the future while taking the correlations between ages and across time into account.

#### Forecasting

For forecasting each coefficient in our FDA model, and for constructing prediction intervals, we use exponential smoothing state space models [9] via the automatic model-selection algorithm of Hyndman et al. [10]. The specific univariate forecasting method is not important, provided that it can handle a variety of time-varying trends.

We use the Mean Integrated Squared Forecasting Error to evaluate the accuracy of the estimated forecasts of future mortalities [3]. This is proportional to the average squared distance between the actual mortality curves and the forecast mortality curves. All statistical analyses were performed in R version 2.10.1.

## Results

**Figure 1:**
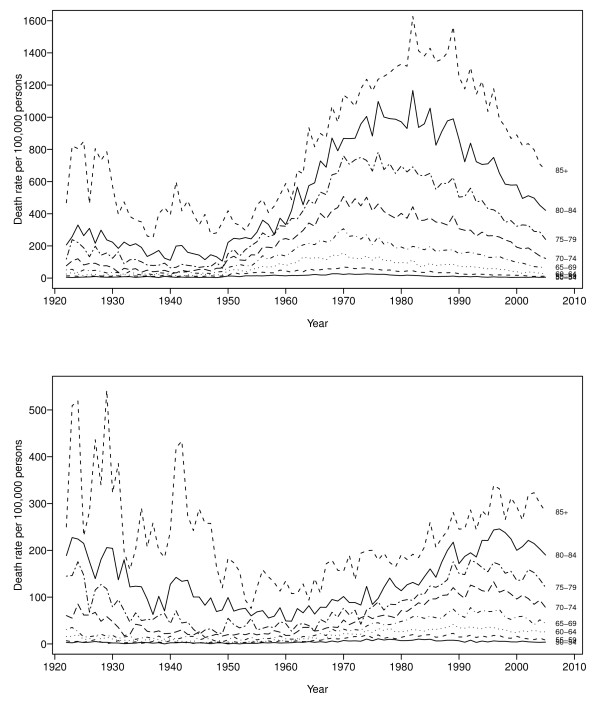
**Age-specific COPD mortality rates in Australia for men (top) and women (bottom) separately, 1922-2005**.

Figure 1 shows the Australian COPD mortality rates for five-year age groups between the ages of 50 and 85+ years over the period 1922-2005. The top panel shows the mortality rates for men and the bottom panel shows the mortality rates for women. The COPD mortality trends for men first began to rise during the 1950s, with the maximum mortality rates for the age group 80-84 being in 1980. They were at their peak during the three decades from the beginning of the 1970s to the end of the 1990s. The COPD mortality trends for women began to increase in 1960 and then peaked in the late 1980s to early 1990s.

In the figure, the rates are simply treated as a collection of discrete observations, without any regard to the underlying probabilistic models. Important features of this time plot are the nonlinear pattern and the evidence of a non-constant variance. The observational error is also clear (as the lines are not smooth), as is the increasing variance for higher ages in the mortality data, particularly in the early part of the century. Clearly, any forecasts of these data will need to be able to model the complex dynamic behavior observed.

**Figure 2:**
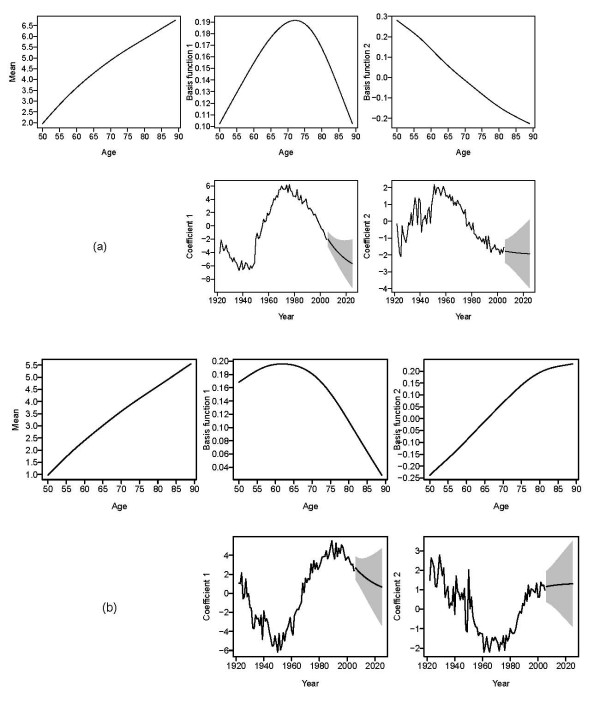
**20 year forecasts of the first two coefficients using state space exponential smoothing models with additive errors and a damped trend for (a) men and (b) women. **The shaded region represents 80% prediction intervals.

Model diagnostics suggest that a set of *k *= 4 basis functions is adequate for both the male and female mortality rates. These four basis functions for men explain 89.9%, 8.3%, 1.2% and 0.4% of the total variation, respectively, and the figures for women are 80.6%, 13.9%, 4.3% and 0.8%. Figure 2(a) and 2(b) shows twenty-year forecasts of the coefficients associated with the first two basis functions, along with 80% prediction intervals, for men and women respectively. Trends in COPD mortality rates among men have been declining since 1970 and the rates are expected to continue to decline for the next 20 years. Likewise, the trends in COPD mortality rates among women are expected to decline, but at a lower rate than that of men. The wide prediction intervals in the mortality forecasts for women suggest a considerable degree of uncertainty in the strength of this trend.

**Figure 3:**
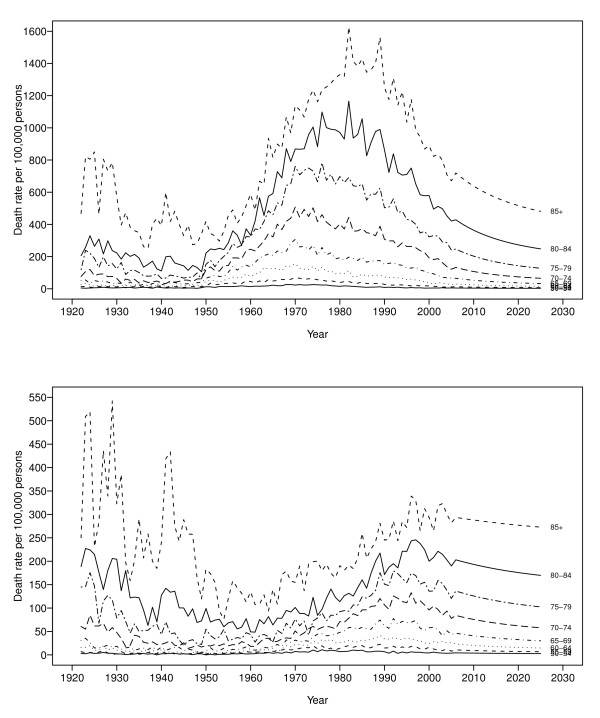
**Forecasts of age-specific COPD mortality rates for the years 2006-2025, for men (top panel) and women (bottom panel)**.

Figure 3 displays the 20-year forecasts by age for men (top panel) and women (bottom panel). For men, the forecasts for the next 20 years suggest a decline in the rate of change of mortality for all age groups, ranging from a decline of 37.1% among those 50 to 54 years of age to a decline of 47.8% among those 75 to 79 years of age. We expect slower rates of decline in COPD mortality for women. For example, we expect the rate of decline to be 32.0% among women from 50 to 54 years of age over the next 20 years, and 21.7% among those 75 to 79 years of age.

**Figure 4:**
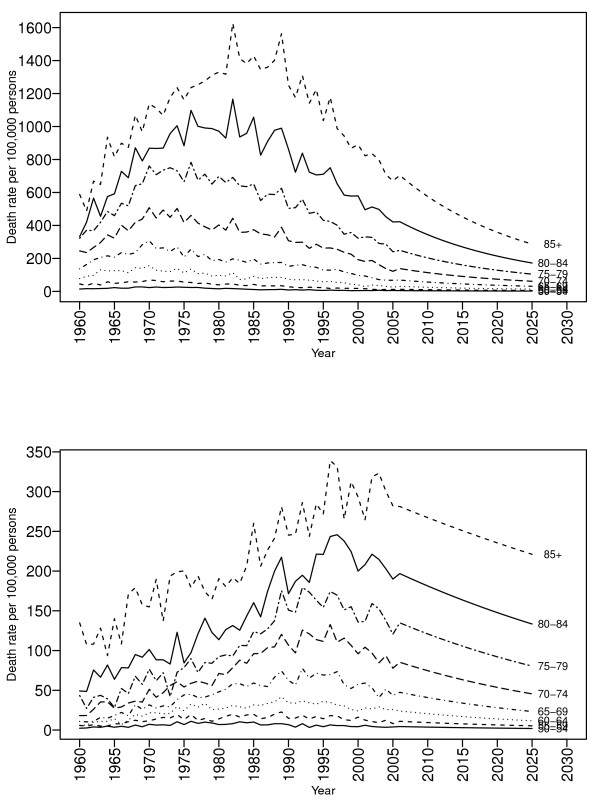
**Forecasts of age-specific COPD mortality rates for the years 2006-2025 excluding data before 1960, for men (top panel) and women (bottom panel)**.

We conducted a sensitivity analysis excluding data before 1960 and refitted the models for the period 1960-2005. For the sensitivity analysis the scale of coefficients were changed from centered on zero to recentered for both male and female The estimated set of k = 4 basis functions did not change. In addition, there was very little difference in the estimated 20 year forecasts by age for men and women compared to 20 year forecasts from the original models [See Figure 4].

## Discussion

To the best of our knowledge, this is the first study to incorporate the effects of age-specific time trends on COPD mortality separately for men and women when estimating future trends in COPD mortality. Our findings suggest that overall COPD mortality trends will continue to decline, but that this decline will be later for women than for men.

As our focus was on comparing the COPD mortality rates of men and women, we did not compare our overall COPD mortality trends with those found in other studies directly. However, our findings on the decline of COPD mortality rates among men are in agreement with studies in many other countries. Using an age adjusted trend analysis, M.J. Goldacre and colleagues [11] demonstrated an overall decline in COPD mortality for men in England over the period 1979-1998. When stratified by age, they also found a decline for all age groups. An earlier study of COPD mortality trends in Canada for the period 1951-1995 [12] reported a decline in the overall COPD mortality for men, with a decline in the 65-74 year age group and an increase among men over 75 years of age. In the United States (USA), COPD mortality rates increased from 1968 to mid 1980s, [13] stabilized, and then declined after 2000 [14]. Similarly, the mortality trends for men in France began to decline steadily after 1985, and further declined between 2000 and 2002 [15].

Our findings on increasing COPD mortality trends for women pre-1997 parallel the patterns observed in studies in other industrialized countries. However, our findings on the decline over recent years are not in agreement with these studies, as recent data are not yet available for comparison. In the United Kingdom (UK), rates for women continued to increase between 1978 and 1998, with the greatest increase being in women over 65 years of age. This finding was consistent across the different combinations used to code the underlying cause of death [11]. We do not have more recent UK data for a comparison of trends beyond 1998. Similarly, COPD mortality rates for women in Canada continued to increase from the late 1950s to 1995, with an increase in age-specific mortality rates in women aged 65 years and over [12]. More recent data from the United States suggest that COPD mortality rates among women tripled between 1980 and 2000, and in 2000 were higher than the rates in men [14]. Our findings are in agreement with a study from France (where recent data were available) which found that female mortality rates increased slightly between 1979 and 1999 and declined thereafter [15].

The difference in mortality trends between men and women may be explained by a number of factors. The main causal factor is tobacco exposure. COPD mortality trends in men first began to rise during the 1950s, with the maximum death rates for the age group 80-84 in 1980. The trends in COPD mortality rates among men mirrored the pattern of the uptake of smoking 50 years earlier. By 1945, almost 72% of men were smokers. Smoking rates among men then fell sharply during the 1950s and early 1960s when concerns about effects on health were first raised [16,17], but, as a result of the aggressive marketing of tobacco products to younger smokers in the 1960s and 1970s, subsequently remained stable until the early 1980s and the advent of organized Quit campaigns.

Similarly, the patterns of COPD mortality trends among women have reflected the patterns of smoking uptake among women. In the years leading up to and during World War II, the advent of movies depicting glamorous images of women smoking, the entry of women into the workforce, and the development of cigarette brands which were attractive to women resulted in widespread uptake, with more than one in every four women reported as smoking by 1945 [18].

While many women also quit in response to the health concerns raised in the later 1950s and early 1960s, the effects of this were offset by increased uptake by women who were born in the 1950s and 1960s, corresponding to aggressive marketing by the tobacco companies, and the increased economic and social freedom of women since that time. Smoking rates therefore barely changed until the late 1980s, but have been declining steadily since 1998 [19].

In addition, these effects may be prolonged by the action of other risk factors such as genetics, indoor and outdoor environment, and lifestyle [20,21]. Other factors include the possible biological and physiological differences between men and women [22]. Women seem to be less likely to be diagnosed with COPD (even with severe disability), and more likely to be misdiagnosed with asthma than men. Chapman and colleagues [23] found that physicians were less likely to diagnose COPD in women (49%) than in men (64.6%), where both presented with hypothetical cases of cough and dyspnea. Women may also differ from men in their response to treatment and disease management [24]. All of these factors subsequently diminish the quality of life with a longer survival time.

Comprehensive tobacco control policies and the decline in tobacco consumption since the early 1970s may explain a considerable component of the declining trend of COPD mortality in women in Australia. As is discussed above, this trend has not been observed in many other countries with a similar population-mix. This may reflect the earlier decline in tobacco consumption in Australia, compared to countries such as the UK and the US; and the considerable success of Australia's QUIT Campaign, compared to smoking cessation strategies in other countries.

There are few published forecasts of age-specific COPD mortality. Two studies from the Netherlands [20,25] developed a dynamic multistate life table model for modelling the prevalence, mortality and health care costs of COPD. Their simulations suggested an increase in COPD-related deaths for both men and women by 2025. These increases became more pronounced in women than men when estimates of smoking prevalence were factored into the model. It is difficult to compare our forecasts with the forecasts from this study because of the differences in modeling approaches and assumptions. For example, these studies assumed constant age- and sex-specific mortality rates for each of disease severity and smoking class. In contrast, our modeling approach produces forecasts based on the entire functional form of the age-mortality curve over time (although they do not take the intervention effects of smoking behavior into account).

The modeling and forecasting approach adopted in this study has a number of strengths. It has the ability to model the functional form of age-related changes in mortality rates over time and make predictions for specific age groups. This enables us to understand the different patterns of disease progression for men and women in younger, middle and older age groups. It provides important insights into the natural history of COPD, which has implications for policy and program development for at-risk age groups.

A second major strength of our approach is the improved modeling of sudden directional changes or jumps in the trends of an outcome. An earlier study of COPD deaths in Australia between 1993 and 2003, and one which generated a considerable amount of debate, modeled the data using linear regression with autocorrelated errors, but failed to capture the sudden change in COPD mortality rates around 1997 [26]. While it is important to understand the reason for this change in the disease pattern, it is equally important for the models and the predictions which they generate to allow for this structural change. The failure to do this is a common phenomenon in trend studies of disease outcomes such as prevalence, incidence and mortality.

Some limitations should be considered when interpreting the results. Our models and the subsequent forecasts do not take into account trends in tobacco consumption, which remains the most important causative factor for COPD in Australia, or the fact that there is a considerable "lag time" between consumption and COPD mortality. The models also fail to take into account intervention effects such as the commencement of smoking cessation programs. There are also well-recognized limitations of mortality data which we do not incorporate into the models. The ascertainment of COPD is often incomplete, as the diagnosis may not have been made during life [27], or may simply have been overlooked in completing the death certificate. COPD is listed as the underlying cause of death only a minority of the time [28]. There have also been changes in the coding of respiratory deaths in the ICD between the third revision in 1922 and the tenth in 1999. These changes confound any interpretation of long-term trends. At present our modeling techniques do not allow for birth-cohort trends, although our diagnostic tests revealed little evidence of cohort trends, with a negligible impact on the estimated basis functions and forecasts.

The national guidelines for COPD management emphasize the cessation of smoking as the only intervention proven to slow the decline in lung function [29,30]. From the graphs it seems that mortality is declining at a slower rate for older women. This is an important finding because this is probably the result of a cumulative exposure to smoking, passive smoking and other lifestyle variables.

## Conclusions

In summary, this is the first study to model and forecast age specific COPD mortality trends in Australia for men and women separately. The predictions suggest widening disparities between men and women in future, particularly among older women. These data provide further insights into age related changes in COPD mortality. Although cessation programs target all smokers - irrespective of age, these data can be used to inform public health prevention policy about possible age groups that may require additional interventions.

## Abbreviations

COPD: Chronic obstructive pulmonary disease; FDA: Functional data analysis; ICD: International classification of disease; UK: United Kingdom.

## Authors' contributions

All authors read and approved the final manuscript. BE, RH, MA contributed to the conception and design of the study, BE, RH, SU, MS contributed to the design of the analytical methods and analysis and interpretation of data and BE, RH, SU, MA, MS contributed to drafting the article and/or revising it critically for important intellectual content.

## Pre-publication history

The pre-publication history for this paper can be accessed here:

http://www.biomedcentral.com/1471-2288/12/17/prepub
